# Metatranscriptomic analysis of colonic mucosal samples exploring the functional role of active microbial consortia in complicated diverticulitis

**DOI:** 10.1128/spectrum.02431-24

**Published:** 2025-05-22

**Authors:** Brittney N. McMullen, Jeremy Chen See, Samantha Baker, Justin R. Wright, Samantha L. C. Anderson, Gregory Yochum, Walter Koltun, Austin Portolese, Nimalan A. Jeganathan, Regina Lamendella

**Affiliations:** 1 Department of Biology, Juniata College, Huntingdon, Pennsylvania, USA; 2 Wright Labs, LLC, Huntingdon, Pennsylvania, USA; 3 Penn State University College of Medicine, Hershey, Pennsylvania, USA; Lerner Research Institute, Cleveland, Ohio, USA

**Keywords:** metatranscriptomics, gut microbiome, diverticulitis

## Abstract

In this study, we investigated complicated diverticulitis, an inflammatory condition associated with abscesses, fistulas, intestinal obstructions, perforations, and primarily affects adults over the age of 60. Although the exact etiology remains unclear, the gut microbiome has been suggested as a contributing factor. Previous studies have used 16S rRNA gene analysis from patient fecal samples, which is limited to identifying the bacterial communities present. Herein, we employed shotgun metatranscriptomics on 40 patient-matched samples of diseased and adjacent normal colonic mucosal tissues from 20 patients with complicated diverticulitis to gain a more comprehensive understanding of active microbial taxa and gene expression patterns that may be involved in this disease state. Our findings revealed distinct beta diversity and a conglomerate of pathogenic microbiota in the diseased tissues, including *Staphylococcus cohnii, Corynebacterium jeikeium, Kineococcus, Talaromyces rugulosus, Campylobacteraceae,* and *Ottowia,* among others. The adjacent normal tissues were a stark contrast, harboring anti-inflammatory taxa such as *Streptococcus salivarius* and housekeeping genes and pathways such as the ABC-2 type transport system ATP-binding protein. These results align with previous amplicon sequencing studies and provide novel functional insights that may be crucial for understanding the etiology of complicated diverticulitis.

**IMPORTANCE:**

Complicated diverticulitis is a virulent condition with no clear cause other than the association with colonic diverticulosis. We assessed the microbial gene expression in complicated diverticulitis patients using colonic tissue samples, revealing microbes in the diseased tissue known to exacerbate the diverticular condition and to live in extreme places, and microbes in patients’ normal tissue known to maintain normal bodily functions. This functional information is therefore important for understanding what microbial taxa are present and what they are doing. It is possible clinicians could someday harness this information to more effectively treat complicated diverticulitis symptoms. For example, clinicians may suggest dietary changes and prescribe probiotics to increase beneficial bacteria. Clinicians may also prescribe targeted antibiotics or consider the emerging treatment option of fecal transplants in complicated diverticulitis patients. While not curing complicated diverticulitis, each potential treatment option mentioned addresses balancing out dysbiosis of the gut microbiome, therefore alleviating associated symptoms.

## INTRODUCTION

Diverticulitis is an acute inflammatory condition of the diverticula usually in the sigmoid colon causing abdominal pain, fever, nausea, vomiting, rectal bleeding, and changes in bowel habits (1). When diverticular inflammation remains within the bowel wall, patients have the sub-classification of uncomplicated diverticulitis, which usually spontaneously resolves but may require antibiotics. When the presence of an abscess, fistula, intestinal obstruction, perforation, or peritonitis occurs in addition to the acute inflammation, the condition is termed complicated diverticulitis, which may be life-threatening and will commonly require surgery (1). Diverticulitis can affect 5%–15% of the general population, although the asymptomatic precursor condition, diverticulosis, characterized by outpouchings of the colon wall, affects more than 60% of individuals over the age of 80 (1, 2).

The mechanisms causing diverticulitis are poorly understood, and there is no definitive biological explanation for the progression of the diverticulitis disease state. The gut microbiome, or all the microbes that reside in the gastrointestinal tract, has been implicated in prior studies. The alteration of the gut microbiota, otherwise known as dysbiosis, compromises the body’s ability to maintain homeostasis (i.e., regulation of inflammatory responses, gut motility, food metabolism, etc.) and may drive disease and allow chronic inflammation to persist (3). Due to the size and sensitivity of the gastrointestinal tract, exacerbation and severity of various inflammatory gastrointestinal diseases, such as inflammatory bowel disease, encompassing ulcerative colitis, Crohn’s disease, and diverticular disease, have been implicated by dysbiosis (3, 4).

The microbiota of 22 complicated diverticulitis abscesses was previously characterized with the enrichment of bacterial taxa, including *E. coli, Streptococcus spp., Bacteroides spp., Peptostreptococcus*, and *Clostridium spp*. (5). It was shown that diseased diverticulitis tissue was enriched in *Microbacteriaceae* and *Ascomycota* taxa, and its adjacent, non-diseased diverticulitis tissue was enriched in *Pseudomonas* and *Basidiomycota* taxa (6). Mucosa samples from the colon of diverticulitis patients were processed with 16S rRNA gene amplicon sequencing and revealed a higher abundance of sulfur-reducing bacteria (SRB) in complicated diverticulitis diseased tissue compared to their adjacent normal tissue (7).

The few microbial investigations into diverticulitis published to date have focused on the sequencing and analysis of the 16S rRNA gene (6–10). This amplicon sequencing technique is limited to showing only what bacterial taxa are present (including taxa in dormancy) but does not divulge which taxa are transcriptionally active and what functions/metabolic pathways they are expressing.

To address this gap in research, this current study utilized shotgun metatranscriptomics on 40 matched diseased and adjacent normal tissues from 20 complicated diverticulitis patients. This study aimed to better elucidate the differential active metabolic pathways endowed by the gut microbiome and how they may be involved in diverticular disease.

## MATERIALS AND METHODS

### Patient demographics

There were 20 complicated diverticulitis patients selected for metatranscriptomic analysis. Ages ranged from 38 to 75, with the average age being 55. BMIs ranged from 20 to 59, with the average BMI being 31.4. There were 8 males (40%), 12 females (60%), 4 ex-smokers (20%), 7 current smokers (35%), and 9 patients who have never smoked (45%).

### Sample collection and RNA extraction

The study was performed under Institutional Review Board approval (#HY98-057EP-A). At the time of surgery at Penn State Hershey Medical Center, patients diagnosed with recurrent, complicated diverticulitis gave informed consent to have surgically resected matched colonic mucosal biopsies collected and stored in the Carlino Family Inflammatory Bowel and Colorectal Diseases Biobank at Penn State Hershey Medical Center, as described in Portolese, et al. (7). Briefly, all patients received the Nichols-Condon bowel prep the day before surgery, which included a laxative and oral antibiotic (11). Samples were harvested from both the diseased tissue (*n* = 20), where the characteristic factors indicating complicated diverticulitis were located, and from adjacent normal tissue (*n* = 20) near the site of diseased tissue. Samples underwent RNA extraction using the ZymoBIOMICS DNA/RNA Miniprep Kit (Zymo Research, Irvine, CA) following the DNA and RNA Purification protocol according to the manufacturer’s instructions with some modifications. Briefly, 50 µL of DNA and RNA were eluted in DNase/RNase-free water and subjected to additional steps of in-column DNase I treatment prior to RNA purification. RNA extracts were stored at −80 °C until further processing.

RNA library preparation was performed on RNA extracts using the NEB Next Ultra II RNA Library Kit for Illumina, following the manufacturer’s instructions (New England BioLabs, INC, Ipswich, MA). This library kit included an rRNA depletion step at the start of the protocol, where a master mix of diluted RNA, hybridization buffer, and rRNA depletion solution was mixed and heated in a thermocycler at 95°C for 2 minutes and ramped down to 22°C. The thermocycler was held at 4°C before proceeding to the RNase H Digestion step. Upon completion of the library kit protocol, equimolar ratios of each library were pooled and gel-purified using 2% agarose gels and GelSTAR Nucleic Acid Stain (Lonza Bioscience, Walkersville, MD) using the QIAquick (QIAGEN, Germantown, MD) gel purification kit. The final purified library quality was assessed using the High Sensitivity DNA Assay on a Bioanalyzer 2100 (Agilent, Santa Clara, CA) before being sequenced in two runs; first on an Illumina HiSeq 4000, generating PE150 reads (UC Davis, CA), and samples with low sequence counts were rerun on an Illumina NovaSeq 6000, using a SP300 flow cell to generate PE150 reads (UC Davis, CA).

### Bioinformatics analysis of metatranscriptome data

After sequencing, raw data quality was assessed for possible errors via generated FastQC (12) reports, and fastp (13) was run to filter reads based on quality. Specifically, a sliding window filter of 4 bases long with a minimum average Q score of 20 was used, and any sequences with fewer than 75 base pairs were removed. Following read filtration, the Kraken2 program (14) assigned National Center for Biotechnology Information taxonomic identifiers to the samples’ sequences and generated taxonomy reports that were used to create a table for species-level analysis. To avoid human contamination from influencing analysis, counts associated with *Homo sapiens* in the species-level annotation table were removed prior to downstream analysis. In addition, sequences identified as *Homo sapiens* were removed using the SeqKit grep command (15) prior to functional annotation. Specifically, the Kraken2-assigned NCBI taxonomic identifier for *Homo sapiens* was searched for in the processed fastq files, and sequences with that identifier were removed before pairing and subsequent functional annotation. The PEAR (pair-end read merger) program (16) merged the forward and reverse reads for each sequence to further increase quality before EggNOG-mapper (17) carried out functional annotations of sequences using the EggNOG database. Results were combined in tables listing the Kyoto Encyclopedia of Genes and Genomes (KEGG) Orthologs (KOs) abundance per sample and the taxa contributing to each KO (18). Downstream analysis of both the Kraken2 generated data and the EggNOG data was then carried out using a QIIME2-2020.11 (19) and R-centric pipeline (20).

### Statistical analysis

Diseased (CD) and adjacent normal (CD-AN) complicated diverticulitis tissue were compared by the following metrics: group, sex, smoking status, BMI, and age of diverticulitis onset.

Beta diversity was calculated for each comparison based on CPM-normalized data for both the taxa and KO data sets, and all plots were visualized with view.qiime2.org or R. PERMANOVA tests were performed on generated Bray-Curtis dissimilarity matrices to assess if a significant difference was observed between samples based on categorical metadata. Mantel tests were performed to see whether samples differed significantly based on continuous metadata. Adonis was run on the same matrix to measure how much variation could be explained by continuous metadata.

LEfSe (21) plots (for categorical metadata) were created based on CPM normalized data and filtered to remove unenriched taxa. Microbiome Multivariate Association with Linear Models (MaAsLin2) determined multivariate associations between metadata and microbial meta-omics features (22). Associations across the CD and CD-AN cohorts were considered for genes, pathways, and microbial taxa identified at the species level.

## RESULTS

In all, 39 samples passed filtration, and sequencing revealed there were, on average, 122,126.821 counts per sample (Table S1). The minimum counts per sample were 4,249, and the maximum was 855,005 counts per sample (Table S1).

### Beta diversity analysis of global active microbial gene expression

Statistical analysis comparing beta diversity revealed no significant differences in microbial expression between diseased and adjacent normal tissues. Statistical significance was lacking in both the expressed microbial taxa and genes for the variables Group (CD vs CD-AN) (PERMANOVA, pseudo-f = 0.288, *P* = 0.967; pseudo-f = 0.495, *P* = 0.915), Sex (PERMANOVA, pseudo-f = 1.929, *P* = 0.071; pseudo-f = 1.407, *P* = 0.182), Smoking Status, including current smokers vs ex-smokers (PERMANOVA, pseudo-f = 1.082, *P* = 0.420; pseudo-f = 1.004, *P* = 0.389), current smokers vs those who have never smoked (PERMANOVA, pseudo-f = 0.825, *P* = 0.490; pseudo-f = 1.173, *P* = 0.295), and ex-smokers vs those who have never smoked (PERMANOVA, pseudo-f = 0.875, *P* = 0.441; pseudo-f = 0.631, *P* = 0.684), and BMI (Mantel Test, Spearman’s rho = 0.039, *P* = 0.704; Spearman’s rho = 0.022, *P* = 0.826) (Table S2). Furthermore, no significant clustering was indicated among the metadata cohorts, supporting the lack of distinct microbial grouping (Bray-Curtis Principal Coordinate Analysis).

**Fig 1:**
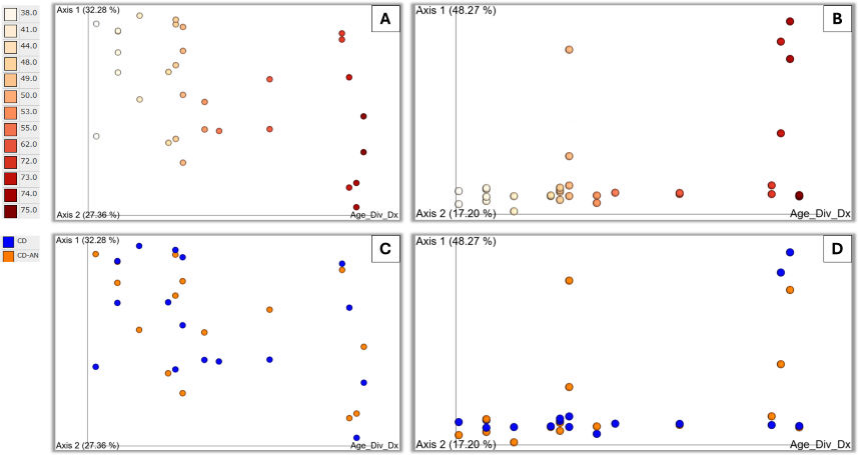
PCoA scatterplots visualizing the age of diverticulitis correlated to expressed genes (A, C) and expressed species (B, D), color-coded by the age of diverticulitis onset itself (A, B) and by group (C, D). Visualization was created on CPM-normalized data and viewed in Qiime2.

The age of diverticulitis onset showed a positive, significant correlation in both the expressed microbial taxa and genes (Mantel Test, Spearman’s rho = 0.15481, *P* = 0.037; Spearman’s rho = 0.194725, *P* = 0.01), where 12.9775% of variation in microbial taxa and 9.4406% of variation in expressed genes was explained by age of diverticulitis onset (Adonis, R2 = 0.129775, *P* = 0.003; Adonis, R2 = 0.094406, *P* = 0.009) (Table S2). This served as an initial indication that greater differences in age of diverticulitis onset were associated with greater differences in overall microbial community and functional gene composition within the samples. Principle coordinate analysis (PCoA) scatterplots visually supported the statistics, showing distinct clustering of both expressed genes and expressed taxa when correlated with age of diverticulitis onset (Fig. 1). Correlations with expressed genes clustered vertically by age of diverticulitis onset itself (Fig. 1A), where younger onset was to the left of the panel and older to the right, but this correlation revealed no distinct clustering based on a group (Fig. 1C). Correlations with expressed taxa clustered tightly by age of diverticulitis onset itself (Fig. 1B), where younger onset ages were to the left of the panel and older to the right. These clusters, especially those of the younger and middle-aged onsets, were tighter than that of Fig. 1A, indicating the expressed taxa of the younger and middle-aged onsets were more similarly related than the genes that were expressed by age. Correlations with expressed taxa clustered loosely by group as well (Fig. 1D), but tighter than those of Fig. 1B. Aside from two samples from CD, most diseased samples clustered loosely horizontally across the bottom of the panel, whereas CD-AN were more scattered (Fig. 1D). This may indicate taxa expressed by diseased tissue are phylogenetically more closely related than the taxa expressed by CD-AN. Furthermore, the taxa that were phylogenetically more closely related were those of younger and middle-aged onsets (the two diseased outliers were 74 and 75 years old at the time of diverticulitis onset) (Fig. 1B and D).

### Actively expressed microbial community structures

While global microbial taxa and gene expression were not significantly different between diseased and adjacent normal tissues, we further investigated if specific microbial taxa were differentially expressed in these cohorts.

**Fig 2:**
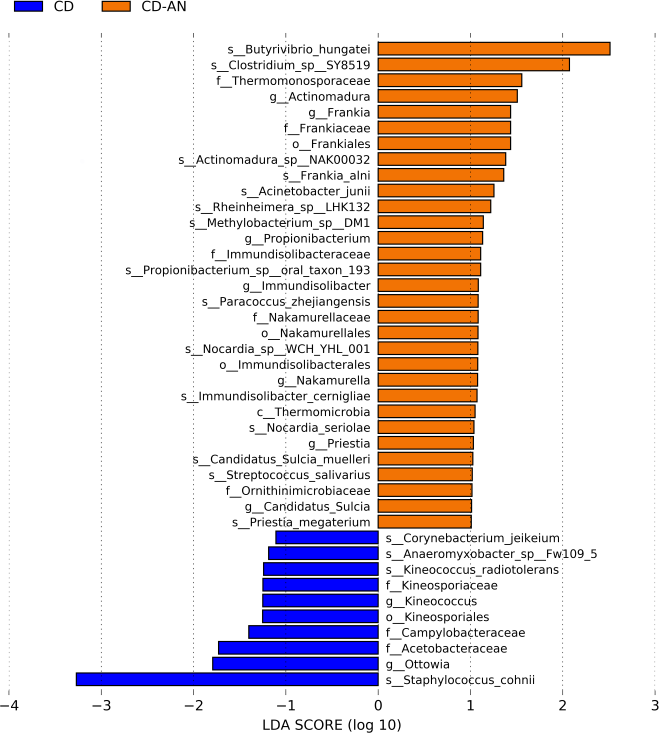
Investigating enriched actively expressed taxa in groups CD and CD-AN. LEfSe bar plot comparing actively expressed enriched taxa in groups CD (blue) and CD-AN (orange), where CD is the diseased diverticulitis tissue and CD-AN is the adjacent normal tissue. The bar plot was created with an LDA cutoff of one.

The CD versus CD-AN comparison revealed more actively expressed taxa in the adjacent normal tissue (31 enriched taxa) than the diseased tissue (10 enriched taxa) (Fig. 2). *Butyrivibrio hungatei* and *Clostridium* SY8519 were the most abundant taxa in CD-AN, though the *Actinomycetia* and *Clostridia* classes, as well as the *Immundicalibacteriales* order, were well represented (Fig. 2; Kruskal Wallis, LDA of 2). In CD, the *Actinomycetia* class was also well represented through the *Kineosporiales* lineage, and *Staphylococcus cohnii* was most abundant (Fig. 2; Kruskal Wallis, LDA of 3). Of the actively expressed taxa enriched, taxa within each cohort appeared phylogenetically related within its respective cohort (Fig. S1).

**Fig 3:**
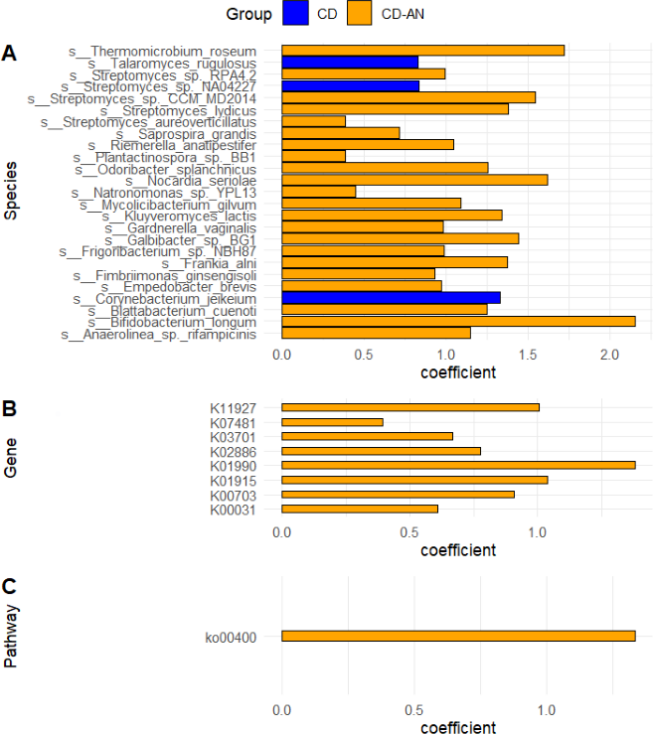
Paneled bar plots visualizing MaAsLin2 determined multivariate associations between the metadata cohorts (CD in blue and CD-AN in orange) and meta-omic features, including (A) microbial taxa differentially identified at the species level, (B) genes differentially expressed, and (C) pathways differentially enriched. The visualization was created in R using the absolute values of MaAsLin2 coefficients.

Multivariate associations between CD and CD-AN and microbial meta-omic features, including genes, pathways, and species, were considered (Fig. 3; Table S3). In all, 8 genes, 1 pathway, and 22 microbial species associations were enriched in the CD-AN cohort (MaAsLin2, positive coefficient, *P* < 0.05) while three species were enriched in the CD cohort (MaAsLin2, negative coefficient, *P* < 0.05) (Fig. 3; Table S3).

Various pathways were tested for expression, but MaAsLin2 reported no statistical enrichment in CD (though visually the Carcinogenesis: ROS, IL-17 Pathway, Drug Metabolism: Cytochrome P450, and Metabolism of Xenobiotics by Cytochrome P450 pathways were enriched in CD).

## DISCUSSION

This study utilized shotgun metatranscriptomics on 40 matched diseased and adjacent normal tissues from complicated diverticulitis patients to better understand the differential active metabolic pathways endowed by the gut microbiome and how they may be involved in the diverticulitis disease state.

No overall significant differences in global microbial expression were observed between diseased and adjacent normal tissues (Table S2). Lacking a true healthy control patient cohort may entertain a level of microbial bias between the matched samples that may limit significant microbial expression, but resecting adjacent normal tissues in lieu of a separate healthy control patient cohort has been the standard research practice of diverticular disease and colorectal cancers (6, 23, 24). Previous studies utilizing matched diseased and adjacent normal tissues have found both insignificant beta diversity (6, 23) and significant beta diversity (24) differences between the matched samples, indicating that matched samples have the potential to have significant beta diversity despite having overlapping microbial communities.

The significant correlation between the age of diverticulitis onset and beta diversity (*P* < 0.05) suggests that older patients exhibit more similar gut microbial phylogeny compared to younger patients (Fig. 1; Table S2). This observation aligns with existing research indicating that the human gut microbiome changes with age, a process that could influence or reflect the progression of diverticulitis (25). As diverticulitis predominantly affects the older population (2), it raises an important question: Is the alteration in gut microbial expression a direct consequence of the disease, or does it result from the natural aging process? The aging gut microbiome is known to undergo shifts in composition and function, potentially altering the gut environment in a way that predisposes individuals to conditions like diverticulitis (25). Alternatively, the disease state itself could exacerbate or drive changes in the microbiome, creating a complex interplay between age, microbial composition, and disease progression. Further studies are needed to clarify the directionality and causality of these changes, particularly whether they are a precursor to or a result of complicated diverticulitis.

Despite this study lacking overall significant differences in global microbial expression, our study revealed specific microbial assembly and expressed genes that differentiated adjacent normal and diseased tissues, revealing interesting patterns within the microbial landscape. Our taxonomic profiling of expressed microbial taxa identified 31 enriched taxa in the adjacent normal tissue compared to only 10 enriched taxa in the diseased tissue (Fig. 2). Pathogenic, pro-inflammatory microbes predominantly enriched the diseased tissues, many of which are not typical inhabitants of the human gut but are instead associated with hostile environments, including both gram-negative and gram-positive organisms.

The presence of gram-negative bacteria, known for their robust outer membrane that confers resistance to antibiotics and immune responses, suggests a microbial environment more resistant to conventional treatments and host immune defense. Notably, pathogenic gram-positive bacteria like *Staphylococcus cohnii* and *Corynebacterium jeikeium,* typically found in hospital settings and associated with nosocomial infections, were enriched in the diseased tissue (26, 27). This enrichment may reflect an opportunistic colonization by these pathogens in an environment already compromised by disease, where normal microbial defenses are weakened. Furthermore, the detection of *Kineococcus* species, organisms generally found in high-radiation environments, suggests an unusual microbial adaptation potentially linked to the presence of reactive oxygen species (ROS) in the diseased tissue, possibly as a result of the inflammatory response (28, 29). The lower number of biomarker features in the diseased tissue compared to adjacent normal tissue may be attributed to the selective pressures exerted by inflammation, immune responses, and antibiotic treatments, creating an environment where only certain resistant or opportunistic pathogens can thrive. This contrasts with the more diverse and presumably balanced microbiota of the adjacent normal tissue, highlighting the impact of the disease state on microbial expression and diversity (30, 31).

The enrichment of *Anaeromyxobacter*, Campylobacteraceae, *Acetobacteraceae*, and *Ottowia* in complicated diverticulitis (CD) tissues highlights the presence of gram-negative bacteria, specifically from the classes epsilon-, alpha-, and beta-proteobacteria. *Anaeromyxobacter*, typically found in soil, can utilize low oxygen concentrations (32). *Campylobacteraceae* includes both commensal and opportunistic pathogenic lineages, with *Campylobacter* known for causing widespread bacterial gastroenteritis (33). Although *Acetobacteraceae* are also commonly soil-dwelling, two species have recently been implicated as human pathogens, particularly in patients with chronic granulomatous disease (CGD) and peritoneal infections (34). While this study could only identify *Acetobacteraceae* at the family level, their presence suggests an involvement in disease in the colonic environment as well. *Ottowia*, although less well studied, has been predominantly isolated from activated sludge, though one species was recently isolated from a human GI tract (35, 36). The presence of these bacteria, often associated with hostile environments, underscores the pathogenic and potentially opportunistic nature of the microbiota in CD-affected tissues.

The notable absence of anti-inflammatory microbial communities in complicated diverticulitis (CD) tissues, contrasted with their enrichment in adjacent normal tissues (CD-AN), aligns with the inflammatory pathway response. For example, *Streptococcus salivarius*, a bacterial taxon enriched in CD-AN, is known for its role in maintaining homeostasis, regulating inflammation by inhibiting NF-kB pathways in human intestinal epithelial cells, and reducing inflammation in colitis models (37). It is also a pioneering colonizer of the human gut, contributing to the establishment of the gut microbiome from birth (37). Similarly, *Butyrivibrio hungateii* in CD-AN produces butyrate, which inhibits pro-inflammatory cytokines and IFN-γ signaling and also helps repair the intestinal epithelial barrier (38, 39). *Clostridium* SY85, another taxon enriched in CD-AN, produces O-desmethylangolensin (O-DMA), a metabolite of the soy isoflavone daidzein, which has antioxidant, anticancer, antimicrobial, and anti-inflammatory properties, including modulation of pro-inflammatory interleukin-6 (40, 41). The presence of these anti-inflammatory microbes in CD-AN tissues suggests a protective role in preventing inflammatory responses and maintaining tissue health. By contrast, the lack of these communities in CD tissues may contribute to the ongoing inflammation and disease progression observed in these patients.

The juxtaposition between CD and CD-AN cohorts is a hallmark of a dysbiotic gut microbiome. Exacerbated disease and chronic inflammation are symptoms of dysbiosis, and both characteristics describe the physical state of the tissue samples resected from the CD cohort (3). The enriched CD taxa mentioned above (Fig. 2) also support this observation in that they were inflammatory and pathogenic by nature. The GI tract is particularly susceptible to dysbiosis and has been linked to several GI conditions including inflammatory bowel disease, Crohn’s disease, and ulcerative colitis (3). The presentation and location of these conditions are also consistent with complicated diverticulitis, further supporting the notion that gut dysbiosis occurs in complicated diverticulitis patients.

In addition to identifying which microbial taxa were differentially enriched in the CD and CD-AN cohorts, a clear dysbiosis in differential functional expression of features, such as genes they contain, pathways they are involved in, and species that are enriched in a respective cohort were also determined across CD and CD-AN (Fig. 3; Table S3). Most enriched features belonged to CD-AN, having 8 genes, 1 pathway, and 22 microbial species enriched. These features were recognized as housekeeping in nature, proving consistency in the sample cohort enriched in (adjacent normal tissue) and supporting the findings of the differential taxa analysis (Fig. 2). CD was enriched in three species but surprisingly lacked enrichment in genes or pathways. The three species enriched in CD were *Talaromyces rugulosus*, a rare pathogenic fungus, *Corynebacterium jeikeium,* a pathogenic gram-positive bacteria, and *Streptomyces* NA04227, another gram-positive bacteria with little known pathogenicity (27, 42, 43).

Overall reduced microbial gene and pathway expression observed in diseased samples, as observed throughout this study, reflect consistent results in studies of similar diseases. For instance, studies on inflammatory bowel diseases (IBD), such as Crohn’s disease and ulcerative colitis, have demonstrated alterations in microbial communities. This observation usually resulted in reduced gene expression of beneficial microbial species and dysbiosis leading to decreased microbial metabolites that play crucial roles in maintaining gut homeostasis and regulating immune responses (44). Furthermore, microbial dysbiosis in the gut, where the expression of genes associated with anti-inflammatory processes is reduced, can disturb immune function, leading to inflammation and sensitization of the immune system that further exacerbates disease conditions. For example, in colitis, certain microbial strains have been shown to reduce inflammation and repair intestinal barrier function, but the overall gene expression of these beneficial microbes may still be diminished compared to healthy individuals (45). Considering this study’s samples are patient matched, the colitis study above supports the current finding that anti-inflammatory genes may still be expressed in a person with a disease, though the enrichment of expression may be site-specific (CD-AN was enriched in anti-inflammatory genes and CD was not).

### Conclusion

This study has several limitations. First, all patients received the Nichols-Condon bowel prep the day before surgery, including a laxative and oral antibiotic, which is known to impact microbial community structure (11, 46). However, because the antibiotic was taken only the day before surgical resection of the colonic mucosal tissue, the full impact of the antibiotic would not have been reached. It should be expected that some depletion of the microbial taxa could be attributed to this treatment, but because the samples were patient matched, similar depletion would be evident in both CD and CD-AN. The results of this study show depletion of microbial taxa in the CD cohort, but not in CD-AN, indicating the overall microbial dysbiosis may be explained by the disease state and not by the antibiotic alone. Second, there was no true healthy control patient cohort due to the ethics of surgically resecting colon samples from healthy individuals. Matched adjacent normal samples were taken to address this limitation, as is the standard research practice for diverticular disease and colorectal cancer studies (6, 23, 24). Having matched samples also accounted for potential confounding factors as the study lacked information regarding whether patients had comorbidities or not. Lastly, due to RNA degradation in the time between RNA extraction and this current shotgun metatranscriptomic study, as well as many sequences being filtered out to avoid human contamination, multiple samples yielded fewer sequence counts than expected. This study also utilized tissue samples, which generally yield fewer microbial sequence counts than the more traditional fecal samples (47). Regardless of the sequencing depth, our sample size was also relatively small. To account for these low sequence counts, beta diversity, LEfSe, and MaAsLin2 analysis were conducted on CPM normalized data, which were scaled and adjusted for the different sequencing depths. Alpha diversity, or the similarity of diversity within sample cohorts, was consequently not investigated.

To our knowledge, this is the first metatranscriptomics study performed on colonic mucosa tissue, a sample localized to the direct site of the disease. This specificity in the location site provides more confidence that changes seen in the microbiota are directly correlated to diverticulitis itself and not attributed to other issues that could excrete the same taxa as found in fecal samples. Metatranscriptomics elucidated not just which taxa were present in the collected samples (as 16S would provide), but also identified which microbial communities were differentially expressed and what function they were expressing. The CD was overall enriched with microbial communities conventionally foreign to the human body, being pathogenic, pro-inflammatory, and rendering the gut microbiome in a dysbiotic state. Enrichment of anti-inflammatory taxa in CD-AN and lack thereof in CD demonstrated consistency with prior research conducted on the immune response and why the perpetuation of the disease may be seen in CD. Differential functional analyses revealed no enriched genes or pathways expressed by the CD cohort, indicating the enriched pathogens deplete the gut microbiome so virulently that no genes or pathways, pathogenic or benign, could be differentially expressed. Thus, it is implied that complicated diverticulitis is driving reduced gene expression in diseased tissue. Housekeeping genes that maintain daily homeostasis were not expressed as is usually found in healthy individuals in the diseased cohort, leading to a decline in the overall health of the diseased individual as homeostatic functions are no longer fully supported. Immunoregulatory genes associated with anti-inflammatory processes were also reduced, leading to inflammation, sensitivity in the immune system, and exacerbation of disease conditions that perpetuate the complicated diseased state.

## Data Availability

The data used in this study have been deidentified and made available in the National Center for Biotechnology Information (NCBI) Sequence Read Archive (SRA) database at https://www.ncbi.nlm.nih.gov/bioproject/PRJNA1212222, under the accession number PRJNA1212222. Sequences are described and labeled as “metatranscriptomics of Homo sapiens: adult colonic mucosa.”

## SUPPLEMENTAL MATERIAL

Figure S1 spectrum.02431-24-s0001.tif Investigating enriched actively expressed taxa in groups CD and CD-AN. Cladogram showing the phylogenetic relationship of enriched actively expressed taxa in CD (blue) and CD-AN (orange), where CD is the diseased diverticulitis tissue and CD-AN is the adjacent normal tissue. Cladogram was created with an LDA cutoff of 1.

Supplemental materials spectrum.02431-24-s0002.docx Tables S1 to S3.

ASM does not own the copyrights to Supplemental Material that may be linked to, or accessed through, an article. The authors have granted ASM a non-exclusive, world-wide license to publish the Supplemental Material files. Please contact the corresponding author directly for reuse.
