# Supplementary figures and images for: Metatranscriptomic analysis of colonic mucosal samples exploring the functional role of active microbial consortia in complicated diverticulitis
Source: Microbiol Spectr. 2025 May 22;13(7):e02431-24. doi: 10.1128/spectrum.02431-24 (PMC12210889; doi:10.1128/spectrum.02431-24)

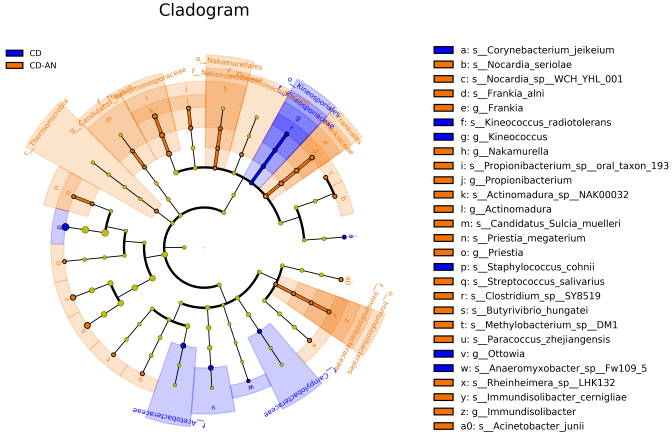

Supplement: Figure S1 — Investigating enriched actively expressed taxa in groups CD and CD-AN. Cladogram showing the phylogenetic relationship of enriched actively expressed taxa in CD (blue) and CD-AN (orange), where CD is the diseased diverticulitis tissue and CD-AN is the adjacent normal tissue. Cladogram was created with an LDA cutoff of 1. [file spectrum.02431-24-s0001.tif]
