# Supplementary material for: Metatranscriptomic analysis of colonic mucosal samples exploring the functional role of active microbial consortia in complicated diverticulitis
Source: Microbiol Spectr. 2025 May 22;13(7):e02431-24. doi: 10.1128/spectrum.02431-24 (PMC12210889; doi:10.1128/spectrum.02431-24)
Supplement: Supplemental materials — Tables S1 to S3. [file spectrum.02431-24-s0002.docx]

# SUPPLEMENTARY MATERIAL

Table S1: Sequencing Count Information

Sequencing summary provided after merging the initial Illumina HiSeq 4000 samples and the Illumina NovaSeq 6000 reruns. Initial number of samples was 40, 20 patient-matched diseased and adjacent normal tissue samples. One sample was removed in filtration due to low sequence counts, leaving a remaining 39 samples for analysis. A total of 3,997 observations were found and a total of 4,762,946 counts were present among all 39 remaining samples. The table summarizes the minimum, maximum, median, mean, and the standard deviation of the counts per sample (‘Counts per Sample - Summary’) as well as provides the sequence count of each sample (‘Counts per Sample - Detailed’).

| **General Sequencing Information** | |
| --- | --- |
| Number of Samples | 39 |
| Number of Observations | 3,997 |
| Total Count for All Samples | 4,762,946 |
| **Counts per Sample - Summary** | |
| Minimum | 4,249.000 |
| Maximum | 855,005.000 |
| Median | 40,762.000 |
| Mean | 122,126.821 |
| Standard Deviation | 170,173.051 |
| **Counts per Sample - Detailed** | |
| *Sample* | *Count* |
| 40 | 4,249.00 |
| 32 | 4,496.00 |
| 31 | 6,044.00 |
| 39 | 6,541.00 |
| 24 | 7,136.00 |
| 37 | 7,191.00 |
| 30 | 7,973.00 |
| 34 | 8,875.00 |
| 36 | 9,456.00 |
| 38 | 10,412.00 |
| 35 | 11,224.00 |
| 29 | 12,181.00 |
| 33 | 12,525.00 |
| 23 | 14,748.00 |
| 25 | 15,213.00 |
| 26 | 18,549.00 |
| 22 | 29,796.00 |
| 28 | 31,092.00 |
| 21 | 36,845.00 |
| 18_Div | 40,762.00 |
| 27 | 40,968.00 |
| 15_265_D | 74,190.00 |
| 16_272_D | 89,151.00 |
| 20_Div | 94,935.00 |
| 15_250_D | 95,999.00 |
| 17_361_D | 105,648.00 |
| 15_250_ND | 164,432.00 |
| 811_157_5_ND | 170,759.00 |
| 811_121_5_D | 197,613.00 |
| 19_Div | 200,189.00 |
| 19_66_ND | 217,980.00 |
| 16_407_D | 230,333.00 |
| 15_249_D | 232,561.00 |
| 16_424_D | 239,668.00 |
| 19_66_D | 242,928.00 |
| 811_157_5_D | 290,501.00 |
| 811_121_5_ND | 390,146.00 |
| 15_265_ND | 534,632.00 |
| 16_424_ND | 855,005.00 |

Table S2: Beta diversity comparisons.

Beta diversity statistical analysis between the group comparison of CD versus CD-AN, sex, smoking status, BMI, and age of diverticulitis onset for both expressed microbial taxa and expressed genes. PERMANOVA, Mantel, Adonis, and p-values were created. p-values ≤ 0.05 were considered significant and were designated above with an asterisk (*).

| **Beta-Significance (categorical)** | | | | | |
| --- | --- | --- | --- | --- | --- |
|  | **Metadata** | **Comparison** | | **PERMANOVA pseudo-F** | **p-value** |
| Expressed Microbial Taxa | Group | CD vs. CD-AN | | 0.288 | 0.967 |
|  | Sex | Female vs. Male | | 1.929 | 0.071 |
|  | Smoking Status | Current Smoker vs. Ex-Smoker | | 1.082 | 0.420 |
|  |  | Current Smoker vs. Never Smoked | | 0.825 | 0.490 |
|  |  | Ex-Smoker vs. Never Smoked | | 0.875 | 0.441 |
| Expressed Genes | Group | CD vs. CD-AN | | 0.495 | 0.915 |
|  | Sex | Female vs. Male | | 1.407 | 0.182 |
|  | Smoking Status | Current Smoker vs. Ex-Smoker | | 1.004 | 0.389 |
|  |  | Current Smoker vs. Never Smoked | | 1.173 | 0.295 |
|  |  | Ex-Smoker vs. Never Smoked | | 0.631 | 0.684 |
| **Beta-Correlation (numerical)** | | | | | |
|  | **Metadata** | **Adonis** | | **Mantel Test** | |
|  |  | **R2** | **p-value** | **Spearman's Rho** | **p-value** |
| Expressed Microbial Taxa | Age of Diverticulitis Onset | 0.129775 | 0.003* | 0.155 | 0.037* |
|  | BMI | 0.059057 | 0.066 | 0.039 | 0.704 |
| Expressed Genes | Age of Diverticulitis Onset | 0.094406 | 0.009* | 0.195 | 0.010* |
|  | BMI | 0.031464 | 0.301 | 0.022 | 0.826 |

Table S3: MaAsLin2 Multivariate Associations

MaAsLin2 determined multivariate associations between the metadata cohorts (CD and CD-AN) and meta-omic features (genes, pathways, and microbial taxa identified at the species level). This table reflects only those associations with a significant p-value (p < 0.05). Cohort associations with a p-value < 0.05 were considered significantly enriched in that feature (gene, pathway, or species), however, no cohort association had a q-value < 0.05. Associations of features enriched in the CD-AN cohort were designated with a positive coefficient while associations of features enriched in the CD cohort were designated by a negative coefficient.

| **MaAsLin2 Significant Multivariate Associations** | | | | | | | | | |
| --- | --- | --- | --- | --- | --- | --- | --- | --- | --- |
| **feature** | **feature identification** | **metadata** | **reference group** | **Co-efficient** | **stderr** | **N** | **N.not.0** | **p-value** | **q-value** |
| Gene | K01915 (glutamine synthetase) | Tissue Resection Site | CD-AN | 1.0413 | 0.424 | 34 | 7 | 0.0197 | 0.7254 |
| Gene | K01990 (ABC-2 type transport system ATP-binding protein) | Tissue Resection Site | CD-AN | 1.3848 | 0.5665 | 34 | 9 | 0.0265 | 0.7254 |
| Gene | K00703 (starch synthase) | Tissue Resection Site | CD-AN | 0.9072 | 0.404 | 34 | 5 | 0.0318 | 0.7254 |
| Gene | K02886 (large subunit ribosomal protein L2) | Tissue Resection Site | CD-AN | 0.7778 | 0.3443 | 34 | 7 | 0.0382 | 0.7254 |
| Gene | K00031 (isocitrate dehydrogenase) | Tissue Resection Site | CD-AN | 0.6074 | 0.2751 | 34 | 6 | 0.0422 | 0.7254 |
| Gene | K11927 (rhlE; ATP-dependent RNA helicase RhlE) | Tissue Resection Site | CD-AN | 1.0053 | 0.4863 | 34 | 5 | 0.0469 | 0.7254 |
| Gene | K07481 (transposase, IS5 family) | Tissue Resection Site | CD-AN | 0.3947 | 0.1879 | 34 | 4 | 0.0491 | 0.7254 |
| Gene | K03701 (excinuclease ABC subunit A) | Tissue Resection Site | CD-AN | 0.6683 | 0.3185 | 34 | 5 | 0.0494 | 0.7254 |
| Pathway | ko00400 (Phenylalanine, tyrosine, and tryptophan biosynthesis) | Tissue Resection Site | CD-AN | 1.3368 | 0.6175 | 34 | 14 | 0.0458 | 0.9833 |
| Species | s__Frankia_alni | Tissue Resection Site | CD-AN | 1.3739 | 0.4431 | 38 | 25 | 0.0037 | 0.7755 |
| Species | s__Thermomicrobium_roseum | Tissue Resection Site | CD-AN | 1.7186 | 0.5878 | 38 | 11 | 0.0091 | 0.7755 |
| Species | s__Kluyveromyces_lactis | Tissue Resection Site | CD-AN | 1.3371 | 0.5219 | 38 | 7 | 0.0147 | 0.7755 |
| Species | s__Blattabacterium_cuenoti | Tissue Resection Site | CD-AN | 1.2459 | 0.4789 | 38 | 10 | 0.018 | 0.7755 |
| Species | s__Talaromyces_rugulosus | Tissue Resection Site | CD-AN | -0.8289 | 0.322 | 38 | 37 | 0.0191 | 0.7755 |
| Species | s__Bifidobacterium_longum | Tissue Resection Site | CD-AN | 2.1533 | 0.8422 | 38 | 23 | 0.0198 | 0.7755 |
| Species | s__Natronomonas_sp._YPL13 | Tissue Resection Site | CD-AN | 0.4473 | 0.1835 | 38 | 5 | 0.0199 | 0.7755 |
| Species | s__Streptomyces_lydicus | Tissue Resection Site | CD-AN | 1.3811 | 0.5655 | 38 | 24 | 0.0251 | 0.7755 |
| Species | s__Saprospira_grandis | Tissue Resection Site | CD-AN | 0.7141 | 0.3075 | 38 | 5 | 0.026 | 0.7755 |
| Species | s__Corynebacterium_jeikeium | Tissue Resection Site | CD-AN | -1.3245 | 0.5723 | 38 | 7 | 0.0265 | 0.7755 |
| Species | s__Nocardia_seriolae | Tissue Resection Site | CD-AN | 1.6162 | 0.7064 | 38 | 20 | 0.0281 | 0.7755 |
| Species | s__Streptomyces_sp._RPA4.2 | Tissue Resection Site | CD-AN | 0.9936 | 0.4255 | 38 | 13 | 0.0313 | 0.7755 |
| Species | s__Galbibacter_sp._BG1 | Tissue Resection Site | CD-AN | 1.4413 | 0.6414 | 38 | 9 | 0.0374 | 0.7755 |
| Species | s__Streptomyces_sp._CCM_MD2014 | Tissue Resection Site | CD-AN | 1.5417 | 0.7151 | 38 | 33 | 0.0378 | 0.7755 |
| Species | s__Fimbriimonas_ginsengisoli | Tissue Resection Site | CD-AN | 0.9275 | 0.4315 | 38 | 8 | 0.0384 | 0.7755 |
| Species | s__Odoribacter_splanchnicus | Tissue Resection Site | CD-AN | 1.2505 | 0.5652 | 38 | 10 | 0.0401 | 0.7755 |
| Species | s__Gardnerella_vaginalis | Tissue Resection Site | CD-AN | 0.9788 | 0.4598 | 38 | 5 | 0.0402 | 0.7755 |
| Species | s__Empedobacter_brevis | Tissue Resection Site | CD-AN | 0.9695 | 0.4571 | 38 | 5 | 0.0409 | 0.7755 |
| Species | s__Anaerolinea_sp._rifampicinis | Tissue Resection Site | CD-AN | 1.1434 | 0.5247 | 38 | 11 | 0.0428 | 0.7755 |
| Species | s__Mycolicibacterium_gilvum | Tissue Resection Site | CD-AN | 1.0877 | 0.5009 | 38 | 9 | 0.0435 | 0.7755 |
| Species | s__Streptomyces_sp._NA04227 | Tissue Resection Site | CD-AN | -0.8352 | 0.3878 | 38 | 15 | 0.0451 | 0.7755 |
| Species | s__Riemerella_anatipestifer | Tissue Resection Site | CD-AN | 1.0432 | 0.5047 | 38 | 5 | 0.046 | 0.7755 |
| Species | s__Frigoribacterium_sp._NBH87 | Tissue Resection Site | CD-AN | 0.9864 | 0.4822 | 38 | 5 | 0.0481 | 0.7755 |
| Species | s__Plantactinospora_sp._BB1 | Tissue Resection Site | CD-AN | 0.3828 | 0.1888 | 38 | 4 | 0.05 | 0.7755 |
| Species | s__Streptomyces_aureoverticillatus | Tissue Resection Site | CD-AN | 0.3828 | 0.1888 | 38 | 4 | 0.05 | 0.7755 |
